# The Control of Private Hospitals

**Published:** 1913-11-01

**Authors:** 


### The Hospital

The Workers' Newspaper of Administrative Medicine and Institutional Life, Administration, National Insurance and Health.

No. 1427, Vol. LV. SATURDAY, NOVEMBER 1, 1913. PRICE ONE PENNY.

### THE CONTROL OF PRIVATE HOSPITALS.

At a meeting of the London County Council on October 21 a resolution was passed by an absolute majority of the whole Council to seek in the Parliamentary Session of 1914 for powers in respect of the regulation of lying-in homes or institutions. The legislation contemplated will confer on the Council power to compel the registration of all premises used for the reception of women for confinement and lying-in, other than Poor-Law institutions, hospitals recognised by the Central Midwives Board, private residences in which the occupier receives a relative, and recognised hospitals to be defined by schedule or otherwise. The Council's officers are to have the right of entry into registered premises and into premises suspected of being so utilised; and the Council is to have power to refuse to grant or to renew a licence, or summarily to withdraw one, if the premises are not suitably equipped or if the licensee is held not to be a fit and proper person. It is also announced that the Council is to consider shortly the promotion of similar legislation dealing with nursing homes and other establishments (not being public hospitals) where massage, manicure, or curative treatment by light, electricity, baths, or similar means is carried on.

It is well pointed out in the *Lancet* that this projected registration and control involves much more than might at first sight appear. Ostensibly these powers are sought in order to assist the police in the suppression of disorderly houses masquerading under the various guises we have enumerated, and to control lying-in homes whose conduct is questionable, if not actually criminal in nature. With these objects every decent citizen must surely sympathise. Yet we may well pause a moment to scrutinise closely the sweeping measure of authority which the London County Council seeks to acquire for itself. The improvement of the standard of efficiency, the registration and inspection of nursing homes and private therapeutic establishments of all kinds has always been strongly advocated by The Hospital, and no carefully considered measure which will achieve these ends will lack such support as we can give it. But we agree with the *Lancet* that it is impolitic and unwise to attack the problem piecemeal and without full discussion of all its aspects. Licensing and registration such as is proposed by the County Council will perforce apply, not only to private homes and hospitals of doubtful moral character or of unqualified quack practice, but also to the very large number of perfectly genuine and respectable establishments which have been equipped of late years to satisfy a public demand. That this is so will be evident when it is remembered that medical men frequently prescribe massage, electrical treatment, and the like for patients in nursing homes; and that many ladies now enter such homes for the purpose of their confinements. In other words, all private homes of every class will have to register and be licensed, and their professional equipment and efficiency are to be adjudged by such officials as the London County Council may appoint for the purpose.

The powers sought are, to our thinking, too wide for a local authority to exercise with advantage. A scheme for improving the standard of genuine private hospitals, large or small, should not be local, but general; otherwise the administrative County of London may find itself fringed by a ring of unregistered establishments in Middlesex, Surrey, Kent, and Essex, just beyond the boundaries of its jurisdiction; and the evils which it is sought to abate will merely have shifted their locality. Before committing itself to any legislation of this nature, Parliament will do well to take a broader view of the matter than a local authority, however excellent its intentions, can be expected to entertain. The suppression of houses of ill-fame trading as establishments for massage, baths, and so on is, of course, highly desirable. If the police find themselves hampered in this aim by defects in the law, let their hands be strengthened by all means, so that they can carry it out more effectually. To take this function out of the hands of the police and transfer it to local authorities seems to us an error; and at least it is a policy which should be fully debated in all its aspects, national as well as parochial. The regulation of quack temples purporting to cure all and sundry diseases is also a worthy object; but, again, we doubt whether a County Council is the most suitable authority for undertaking such a winnowing of wheat and chaff. As for the determination of the quality of the nursing, equipment, and general conduct of private homes, whether for maternity work or for general cases, we feel very strongly that only to a tribunal of experienced medical men and trained nurses of high status can such functions be usefully assigned; and we trust that legislation will be postponed until all these complicated questions have been threshed out by the best intellects that can be brought to bear upon them.

